# Treatment and help services for gambling during COVID-19: Experiences of gamblers and their concerned significant others

**DOI:** 10.1177/14550725211030727

**Published:** 2021-08-12

**Authors:** Virve Marionneau, Johanna Järvinen-Tassopoulos

**Affiliations:** 3835 Helsingin yliopisto, Helsinki, Finland; 3837 Terveyden ja hyvinvoinnin laitos, Helsinki, Finland

**Keywords:** COVID-19, Finland, gambling, public health, services

## Abstract

**Aims:**

During the COVID-19 pandemic, many treatment or help services for gambling were closed or moved online. At the same time, closures of gambling opportunities impacted gambling availability and practices. This study investigates gamblers’ and their concerned significant others’ (CSOs) experiences and views on treatment and help services during this exceptional time and perceptions on how to develop services further after the pandemic.

**Design:**

Three online questionnaires to elicit gambler and CSO experiences were conducted during the spring 2020 in Finland. In total, 847 respondents answered and shared experiences on how the situation had impacted their gambling behaviour and service needs, how service closures or the moving of services online had impacted them, and how they thought the prevention and treatment of gambling harms should be organised during and after COVID-19.

**Results:**

Changed gambling practices reduced overall service needs. Service closures had negative impacts, but online services were considered positively, as these provided a low-threshold option. Respondents also shared insights into how the service provision for gamblers should further be developed during and after COVID-19.

Gambling causes a variety of individual, social, and societal harms, ranging from ill health and financial issues for individuals and families, to crime, societal costs, and social inequalities (cf. Sulkunen et al., 2019). In Finland, 3% of the population experience gambling problems directly, with an additional 21% of concerned significant others (CSOs) experiencing harm due to the gambling of others (Salonen et al., 2020). The treatment and prevention of particularly individual-level harms is conducted within the realm of social welfare and mental health services, consisting of social work, medical help, screening for problem gambling, peer support, debt counselling, child services, but also preventive work (also Dowling et al., 2017; Johnstone & Regan, 2020; Manning et al., 2020). Most services are aimed at those individuals who gamble at high-risk levels or those diagnosed as problem or pathological gamblers, but professionals are rarely trained specialists in gambling-related issues (Manning et al., 2020).

In Finland, the treatment and help service network entails municipal, state-level and third-sector actors that provide services at three levels: specialised support and counselling services for gambling organised by associations (peer support, information, helpline), specialised treatment services for addictions including gambling (institutional and outpatient care, self-help, peer support), and more general public social and healthcare services (identification of problems, directing to other services). In addition, debt counselling and financial aid are provided by both the public and third sectors (Paavonen & Salminen, 2020). Different services also target varying population groups, such as adults, youth, children, elderly people, and immigrants (Järvinen-Tassopoulos & Kesänen, 2020).

The COVID-19 pandemic has caused changes for gambling since spring 2020. The closure of land-based gambling venues and the cancellation of major sports events to mitigate the risk of infection impacted gambling availability and practices. The Finnish system is based on a full state monopoly on all gambling products, and a particularly wide availability of electronic gambling machines (EGMs) in convenience locations. The monopoly holder Veikkaus started reducing the number of EGMs in early 2020, but Finland will have comparatively high numbers particularly of convenience EGMs even after these reductions (Heiskanen et al., 2020). The most visible COVID-19-related restrictions in the Finnish gambling landscape were therefore the closure of the EGMs.

Research has thus far shown that availability restrictions reduced overall gambling consumption in the Nordics (Auer et al., 2020; Håkansson, 2020; Järvinen-Tassopoulos et al., 2020; Lindner et al., 2020; Taloustutkimus, 2020). However, variations in lockdown policies need to be considered, such as the more lenient Swedish restrictions (Ghaharian & Bernhard, 2020). International evidence suggests that some gamblers have also transitioned to online environments (Lindner et al., 2020; see also Cherkasova, 2020; Gambling Commission, 2020; Responsible Gambling Council, 2020), but shifts online do not appear to have occurred in the Finnish context (Taloustutkimus, 2020; Veikkaus, 2021). Some results from other countries also indicate that problematic gambling behaviour and gambling-related harms have intensified, particularly amongst those who have also experienced anxiety, depression, and excessive alcohol use during the lockdown (Håkansson, 2020; Price, 2020).

In addition to decreased availability of gambling opportunities, COVID-19 lockdowns and social distancing regulations have also impacted treatment and help services for gamblers and CSOs. During spring 2020, some services were closed while others moved online. Statistics from helplines in Ontario (Turner, 2020) and from the Finnish gambling helpline Peluuri (Silvennoinen & Vuorento, 2021) show that reduced gambling availability appears to have also reduced service needs. However, reduced contacts to help services may also result from lesser availability. Already before the pandemic, only an estimated 7–12% of those experiencing gambling problems sought help (Slutske, 2006). In Finland, as elsewhere, those seeking help appear to do so as a last resort when gambling is already causing significant harms to themselves and their CSOs (Järvinen-Tassopoulos, 2018; Paavonen & Salminen, 2020).

The current article investigates gamblers’ and their concerned significant others’ (CSOs) experiences and views on treatment and help services during spring 2020 in Finland, and their suggestions for how services and prevention should be (better) organised during and after COVID-19. We adopt a wide definition of services, including not only treatment and help for those already experiencing gambling harms, but also preventive work. The analysis is based on data collected via online questionnaires (*N* = 847). In the following, we will first describe the data and methods used in the study. Second, we present the analysis focusing on service needs, impacts of service closures or the transfer of services online, and more general suggestions raised by the respondents regarding improvements in the service sector. Finally, we discuss the results and limitations of the study.

## Data and methods

The data were collected with three separate online questionnaires during the spring of 2020 in Finland. The questionnaires were conducted by the University of Helsinki (UH), the SOSPED Foundation that provides help services for problem gamblers, and the Finnish Association for Substance Abuse Prevention (EHYT) which coordinates and operates gambling harm prevention in Finland. These questionnaires were conducted separately, as each instance initially set out to collect data on gambling during the pandemic for their own organisational needs. Each questionnaire was therefore initiated independently but collaboration was then introduced.

The overall aim of each questionnaire was to chart changes in Finnish gambling during the exceptional closures of many gambling opportunities (gambling arcades, EGMs, and the casino) as well as treatment and help services during the spring 2020 COVID-19 lockdown, but with somewhat differing foci. The SOSPED questionnaire aimed at charting the experiences of their customers (help-seekers) on gambling harms and problem gambling during the pandemic. The questions were drawn up in collaboration with SOSPED professionals and the researchers at UH. The EHYT questionnaire was aimed at all respondents, including non-gamblers, and focused on questions regarding changes in consumption patterns and prevention. The questions were drawn up by the EHYT team but researchers at UH were also consulted. The UH questionnaire was aimed at active gamblers and focused more on societal issues. The questions were drawn up by the UH researchers in collaboration with the SOSPED team as well as professionals at the Peluuri gambling helpline. The data were pooled for this analysis to form a wide picture of experiences on gambling services during COVID-19. A total of 847 respondents participated. Of these, 688 responded as gamblers, 97 as CSOs and 62 as both. Problematic gambling of the respondents was not screened.

**Figure 1.**
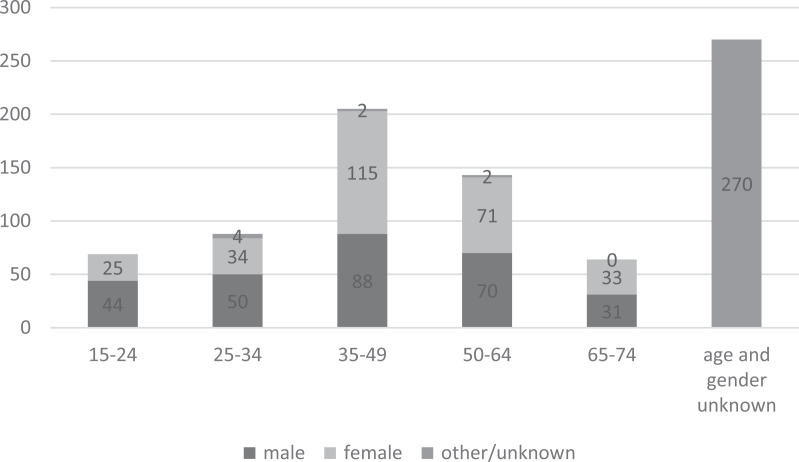
The age and gender distribution of the respondents (*N* = 847).

The age and gender distribution of the respondents is described in Figure 1. The share of those who did not disclose this information is important because the SOSPED questionnaire did not ask the respondents (their customers) such background information. While this is an important limitation, the questionnaire was nevertheless included in this analysis as these respondents were familiar with the treatment and help services and had valuable insight into the question.

The EHYT questionnaire was open between 14 April and 8 May 2020. The UH questionnaire was open between 16 April and 19 June 2020. The SOSPED questionnaire was opened on the 9 April 2020. Data collection from this questionnaire ended on 25 May 2020 when the first results were published. While the questionnaire remained open after the publication of initial results, publication may have impacted subsequent responses for which reason these data are not considered in the current analysis.

Each questionnaire was widely distributed in different online channels, including social media accounts, online communities, forums, websites of treatment and help services, newsletters, and as direct invitations to customers of help and support services. The UH and SOSPED questionnaires were also distributed to help-seekers via the channels of SOSPED and the Peluuri helpline. The EHYT questionnaire was aimed at participants over 15 years of age, while the UH and SOSPED questionnaires were aimed at participants over the age of 18 years.

Each questionnaire consisted of multiple-choice and open-ended questions. The EHYT and UH questionnaires also had background information questions. All questions relating to services, as well as the number of responses for each question, are detailed in Table 1. These are also the questions that have been included in the current analysis. We have therefore excluded questions that were not directly related to services, including questions related to changes in consumption patterns, perceptions of marketing, views on EGM closures, and impacts of the COVID-19 pandemic on family relationships or financial circumstances.

**Table 1.** Questionnaire items related to gambling services and responses.

Question	Responses / total respondents
EHYT questionnaire	
Gamblers and CSOs: What kind of help or support do you need for gambling? Has the need changed during COVID-19?	246 / 452
Gamblers and CSOs: How should gambling harms be prevented during COVID-19?	233 / 452
SOSPED questionnaire	
Gamblers: How should the impacts of COVID-19 be considered in the support and information aimed at gamblers? What kind of services or information would be beneficial to you?	66 / 214
CSOs: How should the impacts of COVID-19 be considered in the support and information aimed at CSOs? What kind of services or information would be beneficial to you?	26 / 52
UH questionnaire	
Gamblers: Have you experienced harm due to closures of help services during the COVID-19?	36 / 122
CSOs: Have you or your gambling family member/friend experienced harm due to the closures of help services during COVID-19?	4 / 7
Gamblers: If you currently need support for your gambling, what kind of support could that be?	21 / 122

*Note*. EHYT = the Finnish Association for Substance Abuse Prevention; CSO = concerned significant others; UH = the University of Helsinki.

The included questions were asked in slightly different ways to both gamblers and their concerned significant others in the SOSPED and UH questionnaires. The EHYT questionnaire was designed to accommodate both respondent groups in the same questionnaire form. None of the questionnaires required a mandatory answer for any of the questions.

As the questions were open-ended, we used qualitative content analysis (Elo et al., 2014). First, the material was read many times for it to be categorised (as themes), abstracted (as codes) and interpreted (as results). The unit of analysis used here is a full answer to an open-ended question. The answers were, on average, one sentence long. In the organisation phase, consisting of categorisation, abstraction and interpretation, we categorised four themes: changed needs for services; impact of services closures; improvement suggestions on help services during and after COVID-19; harm prevention initiative suggestions during and after COVID-19.

The data were subsequently abstracted: a total of 632 responses were coded using Atlas.ti software. In addition to the main categories, we also coded responses that indicated “no opinion” or “no interest” as a separate category for each question. Blank answers were not coded separately but are noted for reference in Table 1. We did not analyse frequencies of open-ended answers as the focus was on the qualitative content of the data. Finally, we interpreted the qualitative data into results by using quotations to highlight the different aspects of our analysis. The quotations are presented with background information of the respondent (gambler/CSO, age group, gender).

Unlike population studies, the online questionnaires did not constitute a representative sample of Finnish gamblers or CSOs. For example, groups such as the elderly may not be able to participate via online channels. Another limitation of these data is that, although the three questionnaires were aimed at differing audiences, some respondents may have answered in several questionnaires. For these reasons, the data were not analysed statistically. At the same time, online questionnaires can constitute a rapid data collection method that is possible to implement even during exceptional circumstances such as COVID-19. While a statistical analysis of the data may not be reliable, the respondents constitute a valuable group to analyse from a qualitative perspective, particularly since the share of gamblers, CSOs of gamblers and those experiencing gambling harms is likely to be higher in this type of questionnaire than in population studies. Online surveys may also represent different groups of gamblers and CSOs and thus they can be used to build groundwork on novel topics that may be expanded later via, for example, interview studies (Kinnunen & Mäyrä, 2014).

## Results

### Changed needs for treatment or help services during the COVID-19 lockdown

Existing literature of consumption changes during the COVID-19 lockdowns in spring 2020 indicate that, overall, total consumption declined, but the consumption of online gambling was maintained at existing levels, or increased in some contexts (Auer et al., 2020; Cherkasova, 2020; Håkansson, 2020; Responsible Gambling Council, 2020; Veikkaus, 2021). Similar changes in consumption were also visible in the questionnaire data. Our previous analysis shows reductions in gambling during COVID-19 across all products, albeit less so for online gambling (Järvinen-Tassopoulos et al., 2020).

The reduced consumption of gambling was also reflected as an overall reduced need for treatment and help services for both gamblers and CSOs during the spring of 2020. The UH questionnaire asked participants whether they had contacted any help or support services during the COVID-19 lockdown, to which only three respondents (of 122) answered yes. Instead, the respondents voiced relief regarding the closures, particularly of EGMs. In previous research, EGMs have been described as the “crack-cocaine” of gambling due to their inherent structural characteristics (Dowling et al., 2005). Gambling harms and diagnosed gambling disorder severity are also positively associated with internet gambling and particularly online EGMs (Gainsbury, 2015). In our questionnaire data, EGM closures were descried as a welcome development, particularly for those who experienced problems with their gambling before the lockdown. The closure of EGMs was also reflected as a reduced or a quenched need for services particularly for those who used to play at EGMs.

I don’t have any need [for services], I’m feeling really good right now. I don’t miss the gambling machines. I hope they would be permanently moved from public spaces to arcades. (Gambler, male, 35–49, EHYT)

Respondents also expressed a need for additional or improved services to maintain the positive effects of the gambling closures. One participant wondered whether they could “keep walking past the machines after this dry spell” (gambler, male, 35–49, EHYT) while another noted that help services would particularly be needed to maintain this newly found gambling-free lifestyle:

It would be good to have widely available distance support right not. Now many have almost a mandatory opportunity to wean off gambling, and correctly aimed support might be crucial to also not gamble in the future. (CSO, gender and age unknown, SOSPED)

Another issue that respondents identified as a cause for increase service needs was the possible shift towards online gambling or increased gambling participation of online gamblers. One participant described how their own gambling with EGMs had reduced, but they were “concerned for online gamblers, because they have more time and they can’t go out as much. Online gamblers have a lot of need for support” (CSO, gender and age unknown, SOSPED). Online help services may also be problematic during these circumstances. A recovering problem gambler described the difficulties in doing everything online where gambling opportunities are also constantly present:

If there are resources, a discussion group via video might work, but it also has its limits: after the session you would still be on [the internet] and games would only be a click away. (Gambler, gender and age unknown, SOSPED)

Changes in the forms and availability of services could therefore also be considered an issue that might prove problematic for some as described in the following section.

### Impacts of service closures and the transfer of services online

Responses elucidating the impacts of changes in services were divided into two main groups. The first group consisted of those respondents whose usual help and support services were discontinued during the COVID-19 lockdown or who were unsure how services would be organised under the lockdown period. Particularly CSO respondents were concerned over changes or closures and how these might affect their gambling close one. These respondents had experienced increased harms due to this situation and considered that support has been particularly weak:

[Service needs] have changed, because my CSO’s visits to the psychologist were cancelled. The support has been really bad. (CSO, female, 25–34, EHYT)

The second category of responses related to services moving online. For some, these online meetings have not been as useful as live meetings. However, others noted that they are gradually getting used to meeting online:

My support group has become a video conference. It’s a bit different than meeting face-to-face, but it is slowly starting to take form. I’m grateful that we can meet at distance and [the group has] not been obliged to close. (CSO, female, 39, UH)

Some participants also noted that the online environment provides additional channels for keeping in touch, such as “closed weekly groups on WhatsApp” (gambler, female, 50–64, EHYT) or getting peer support via email or messaging. Regular messages were considered particularly useful to maintain abstinence from games:

During the lockdown, it’s a good idea to send messages of support to those with problems, and to remind them that it’s a good time to start recovery. Particularly if EGMs are a big problem. (Gambler, gender and age unknown, SOSPED)

### Suggestions for improving existing help services during and after COVID-19

Ideas for improving existing help services were divided into COVID-specific suggestions and suggestions for more long-term changes in service provision. Suggestions for improvements came mainly from gamblers. This may be related to the fact that gamblers have more first-hand experience of help and treatment services than CSOs.

The new online tools, such as regular support messaging, were considered potentially useful to preserve after the lockdowns to make services better and more widely available. Help services should be easily accessible and easy to find through, for example, “active visibility on social media” (gambler, female, 35–49, EHYT). Maintaining the new low-threshold services such as online interventions, online chats, moral support, or “even small contacts such as this questionnaire” (gambler, gender and age unknown, SOSPED) found support.

Nowadays I have been able to control my gambling, but before I would have benefitted most from a support service that has a low threshold to seek help. So that you would not immediately be labelled as a problem gambler, because it might be difficult to admit to yourself and you might not notice it yourself before you owe several thousands to instant loan companies. (Gambler, male, 24, UH)

Less related to the COVID-19 situation, the respondents also called for more “sensitivity and courage from professionals to bring up the topic” (gambler, female, 35–49, EHYT), or the “integration of gambling addiction support in occupational healthcare” (gambler, male, 30, UH) to improve service availability in the future. Treatment and counselling for gambling-related issues are concentrated in the third sector in Finland, while the public sector has a reduced role (Paavonen & Salminen, 2020). Based on the examples given by the respondents of the current study, this appears to mean that, while services for gambling-related harms do exist, they are not integrated fully on the agenda of public service providers from health professionals to social workers. Another issue in finding adequate specialised services is that they may not be available locally (Järvinen-Tassopoulos & Kesänen, 2020). Gambling-related services therefore need to be specifically sought out rather than integrated into the agenda of services such as health check-ups, the social benefit system, income support, and child services. This reduces the possibilities of the service sector to bring up the topic early on and to initiate early interventions.

The kind of support that respondents who have had first-hand experience with gambling problems as particularly important were “psychological support for problem gamblers or potential problem gamblers” (gambler, male, 25–34, EHYT), regular visits with treatment specialists or peer groups, as well as professional debt management help, such as “social workers negotiating with banks to get affordable loans to those in trouble” (gambler, female, 35–49, EHYT). In addition to professional help, peer support and support person contacts were also considered important and worth improving on during the lockdowns but also in the long run:

[What would be needed is] that after this lockdown different cities would have more peer support groups, and that gambling problems in general would be discussed more and more openly, and more support services would be available. For example, one-on-one chats with a recovered problem gambler would in my situation be more than necessary and a great thing if these could be organised. I wish for more resources in the treatment of gambling problems in all of Finland (and that all those who are willing would be accommodated in peer-support groups and other kind of help in this sector). (Gambler, gender and age unknown, SOSPED)

Alongside suggestions for new and improved services, some participants were also already happy with the existing offer. This was particularly the case for those who were happy with the service provision that they were currently getting personally or had received in the past. One respondent noted that while their significant other no longer gambled, “it is comforting to know that you are out there if I ever need help/support/tips” (CSO, gender and age unknown, SOSPED). On balance, other respondents felt that services were non-existent or had previously been disappointed:

There is no support and no help. I once called the gambling helpline, they just talked nonsense. (Gambler, male, 65–74, EHYT)

There are many barriers that may prevent gamblers and their CSOs from seeking help, such as shame, fear of stigmatisation, lack of available services, and an experienced high threshold to seek help (Itäpuisto, 2019; Järvinen-Tassopoulos, 2018, 2020). To make the encounter between professionals and gamblers and their CSOs successful, professionals have been encouraged to be careful not to impose their own values on clients, and not to highlight the responsibility of gamblers for their problems. Instead, focus should be placed on more general processes behind gambling harm, such as structural inequity in society (Järvinen-Tassopoulos & Kesänen, 2020). This is something that should also be considered in service provision during and after the pandemic according to the respondents.

### Improved harm prevention during and after COVID-19

In addition to suggestions for further service improvements, participants also voiced several ideas for improvements in preventing gambling harms either during or after the pandemic. The EHYT questionnaire had specifically prompted respondents on gambling harm prevention during the COVID-19 lockdown and provided particularly many ideas on this. Suggestions for overall prevention of harm fell into four general categories: information campaigns, limit-setting, availability restrictions also after the pandemic, and a public health or welfare policy approach to gambling.

#### Information campaigns

Existing literature on information campaigns as a harm prevention strategy in gambling has not been conclusive about their effectiveness, possibly due to the limited extent to which they have been implemented (see Sulkunen et al., 2019 for a review). Yet, information campaigns were brought up in the questionnaire responses to prevent gambling harms. Respondents had concrete ideas regarding how to improve such campaigns in terms of their content as well as their reach. In terms of content, respondents mentioned that if and when EGMs are reopened, they should have “proper warnings on what gambling can lead to” (gambler, gender and age unknown, SOSPED) and campaigns should focus on “informing about the odds of winning” (gambler, female, 65–74, EHYT). One respondent suggested informing players about how gambling habits have changed during COVID-19 and to reflect on a possibility for a long-term change:

For example, showing by comparison […] that now that the EGMs and gambling opportunities are almost completely closed because of this epidemic and compare one’s own gambling habits [to those before the pandemic], all of ours. In other words, to be able to compare the need to gamble, or whether there is a need. (Gambler, gender and age unknown, SOSPED)

In terms of the possibilities for information campaigns during COVID-19, the participants mentioned online campaigns that would focus on “directly informing problem gamblers about the dangers and addictivity of online gambling” (gambler, gender and age unknown, SOSPED), but also “gaining visibility in social media so that people and information would reach each other” (gambler, female, 35–49, EHYT). On the other hand, many elder people do not use the internet and information should therefore also be available in newspapers or on the television, possibly “including in the news and on special broadcasts about COVID-19” (gambler, gender and age unknown, SOSPED).

#### Limit-setting

In addition to information campaigns, another suggestion to further improve harm prevention during COVID-19 related to limit-setting. Many of the respondents, particularly gamblers, had first-hand experience of using limit-setting as a tool to control gambling, but they also saw room to improve in this respect. During spring 2020, EGMs did not yet require mandatory identification in Finland. Although this reform has been announced as of 2021, some respondents mentioned mandatory limits in land-based gambling, including making the “Veikkaus card mandatory for all, and gambling limits for all” (gambler, male, 50–64, EHYT).

Limitations should be mandatory and based on low daily or weekly limits on spending. The monthly spending limit during the data collection was 2000 euros, which was considered excessive; “a maximum 50-euro limit, it should not be bigger, [the current] 2000 euros is a shocking amount” (gambler, male, 50–64, EHYT). Veikkaus did reduce its monthly and weekly spending limits to 500 euros as of 1 May, 2020 to limit gambling during the pandemic, but the monthly limit was raised back to 2000 euros in October.

Limitations should also be stricter during the COVID-19 period, including “not being able to change your spending limits during the lockdown on Veikkaus or other websites” (gambler, other, 25–34, EHYT), but also limitations on obtaining instant loans. One CSO of a problem gambler described that they would “like to know how to block my CSO from taking on more debt” (CSO, gender and age unknown, SOSPED).

The closure of EGMs due to the pandemic also put focus on limit-setting in online environments. Respondents criticised existing limitation possibilities on both the national Veikkaus platform as well as the websites of offshore providers. Regarding Veikkaus, one respondent noted for instance that “there should be a permanent self-exclusion from Veikkaus games, not just temporary” (gambler, male, 50–64, EHYT). Furthermore, the lack of possibilities for Finnish authorities to regulate offshore providers also reduces or even removes the possibility to self-exclude or set limits in these online environments. “IP blocking and money transfer blocking to foreign sites” (gambler, male, 35–49, EHYT) were suggested as a possible means of reducing gambling harms, but respondents who regularly partook in gambling on offshore websites also criticised blocking as a means to maintain the monopoly system rather than as a means to reduce harms.

#### Availability restrictions

In addition to information and limit-setting, respondents discussed the systemic-level problems of gambling availability in the Finnish monopoly system that was seen to encourage excessive gambling. Respondents across all three questionnaires and both gamblers and CSOs expressed relief following the closure of EGMs, and many wished that the machines would remain shut. The EHYT questionnaire prompted respondents on whether EGMs should be reopened. Forty-one percent of those respondents who answered this questions (*N* = 188) wanted EGMs in convenience locations (supermarkets, petrol stations, restaurants) to remain shut also after the lockdown, while only 26% (*N* = 118) wanted them to reopen. This result may be skewed by the fact that the respondents in the questionnaires were a select group of opinionated individuals. Some support for the finding is nevertheless found in a representative population study (*N* = 1,004) also conducted during the spring 2020 (Taloustutkimus, 2020). The study shows that 32% of respondents wanted to reopen EGMs after the pandemic, while 36% wished for the machines to remain shut.

Even without a lockdown, gambling should be removed from the sphere of everyday life like shops, petrol stations, kiosks, and bars. (Gambler, male, 35–49, EHYT)

Prevention has already been accomplished: gambling machines are closed, casinos are closed, betting has ended. (Gambler, female, 35–49, EHYT)

#### Welfare policy

The reduction of gambling harms was viewed by some as a more general objective of welfare policy than just a question of gambling regulations. As has also been agued extensively in research literature (e.g., Wardle et al., 2021; Wardle et al., 2019), a public health approach to gambling is necessary to shift focus from individuals to populations and better prevent gambling harms in societies. This also means that gambling should not be addressed separately from other population welfare issues. A system such as that in Finland, in which gambling is used to fund a host of health and welfare associations and services (including gambling-related help and treatment services), was considered particularly problematic. While the work of many of these associations was mainly considered important, “the money should be collected elsewhere and more evenly from everybody rather than mainly from problem gamblers” (gambler, gender and age unknown, SOSPED).

In addition to integrating gambling services into the agenda of other state welfare provision, gambling-related harm might also be prevented by improving public health in other areas. Services that would more generally improve “the well-being of the mind” (CSO, gender and age unknown, SOSPED) or “dealing with stress” (CSO, gender and age unknown, SOSPED) would also be beneficial to preventing or reducing gambling harms. Gambling was not always seen as a problem in itself, but rather as a consequence of other problems or feelings of emptiness in life. As one respondent put it, “gambling itself is not a problem for me, but more of a consequence of not having anything else in my life but my work” (gambler, male, 35, UH). As such, the prevention of gambling harms was not necessarily considered to be merely within the remit of specialised associations or service sector actors, but rather of more encompassing approaches promoting individual and social welfare:

To me it seems like the same factors that prevent social exclusion also quite effectively prevent gambling problems. We should focus more widely on people in danger of being marginalised, aim for providing the possibility for everybody to get an education, employment, and social relationships. (Gambler, female, 35–49, EHYT)

## Discussion

The COVID-19 pandemic has had wide-ranging implications, including leaving people without a social safety net and with limited access to healthcare and social services (Jemberie et al., 2020). This has also been true, to some extent, of gambling-related services, although many services have also been modified to be accessible online and in mobile form (Humphrey et al., 2020; Turner, 2020). The results of the current article have shown that, overall, and alongside reduced gambling availability and activity, the need for treatment and other help services for gambling appears to have been reduced. This supports the “total consumption model” or the “availability hypothesis” (Abbott et al., 2016; Lund, 2008; Sulkunen et al., 2019) according to which there is a positive relationship between the number of gambling opportunities, gambling participation, and the proportion of those experiencing harm.

The analysis of gambler and CSO experiences during spring 2020 has also raised three other important conclusions related to service provision that the pandemic appears to have highlighted.

First, while service closures were, unsurprisingly, experienced in a negative light, transfers online were, more surprisingly, considered mainly positively. Particularly psychiatric and addiction care services have made efforts to ensure continuity of care via online means (Jemberie et al., 2020). The low threshold of access to online services was also considered a positive development that should be maintained after the pandemic situation eases. Considering that only a low proportion of gamblers experiencing harm eventually contact help services (Slutske, 2006), a low threshold service network appears to be an important first step towards addressing harms before they develop further (also Paavonen & Salminen, 2020). The finding is also supported by a previous systematic review into the treatments of problem gambling (Petry et al., 2017) which stated that feedback and distance support may be effective at less serious or less advanced problem levels. Low threshold interventions online may therefore be a good practice though which to prevent the escalation of gambling-related problems and harms, and a service option that should also be maintained post COVID-19.

Second, help and treatment services should also be more tightly integrated into wider health, social and welfare service agendas. Finland currently has no national treatment protocol regarding the treatment of problem-gambling-related issues (Paavonen & Salminen, 2020). Services for gambling are encompassing, but also scattered across different public and third sector actors. Particularly non-specialised social workers may lack the expertise to address gambling-related harms (Engel et al., 2012; Järvinen-Tassopoulos & Kesänen, 2020). Some of the respondents in the current study criticised the system, as necessary help was difficult to locate and not proactive. Integrating the identification of gambling-related counselling and treatment in the public service network more tightly might also allow early identification before problems escalate. A recent systematic review of prevention strategies in gambling (Škařupová et al., 2020) found that early identification of gambling problems is connected to better recovery outcomes and lesser harms. The help and treatment sector in gambling problems might benefit from the so-called “coordinated multi-sector strategies and innovative holistic approaches” that are currently more familiar in the substance use sector (Jemberie et al., 2020).

The third conclusion of this study relates to the importance of preventive work rather than treatment of those already inflicted with harm. The pandemic situation has highlighted the importance of harm prevention strategies, including public information on the risks of gambling, mandatory limit-setting, availability restrictions, and marketing bans to limit consumption due to the risk of infection (Järvinen-Tassopoulos et al., 2020). In addition to integrating gambling-related help services into public service provision, the prevention of gambling-related harm could also be integrated into a wider public health agenda. This could be achieved by promoting protective factors against gambling harms, such as social wellbeing and family cohesion (also Dowling et al., 2017).

These conclusions support tackling gambling harms as a public health issue at all levels, not only during the COVID-19 pandemic to limit infections, but also afterwards to limit gambling harms (cf. Wardle et al., 2021; Wardle et al., 2019). If national and local policymakers accept that substance use disorders may require several intervention components, they should accept them also in gambling issues (Jemberie et al., 2020). A public health model addresses not only individual harm, but also the commercial and social determinants of harm, such as availability, accessibility, marketing, and risk factors that also cause harm amongst low and moderate risk gamblers (Griffiths et al., 2009; Johnstone & Regan, 2020). According to the prevention paradox argument, most harm accrues to the low or moderate risk population who also benefit most from interventions (e.g., Browne & Rockloff, 2017). Browne et al. (2017) have found that at a population level, aggregate harms of non-problem gamblers exceeded those accruing to problem gamblers by 4:1. These are similar themes to those raised by the respondents in this study.

The current study has been limited to analysing the experiences of 847 gamblers and CSOs of gamblers during the spring 2020 COVID-19 outbreak in Finland. The data do not constitute a representative sample of the Finnish population and are skewed towards those experiencing harms from gambling. The results cannot therefore be generalised to the whole population. Furthermore, it has not been possible to analyse the data statistically based on, e.g., player profiles. However, from the perspective of investigating the views of gamblers and CSOs on services during and after the pandemic, the sample consisting of many service users has provided rich qualitative data and important insight. As the current study has been mainly exploratory, further studies could focus on the differences between gamblers and CSOs, changes in experiences and views as the pandemic has endured, and on different gambling-related services available during the COVID-19 pandemic. It is especially important to understand how online counselling and mobile services have helped problem gamblers and their CSOs to get by during the pandemic (also Sharman et al., 2021). If online and mobile services continue to exist after the pandemic, further developments are needed to ensure they reach those problem gamblers and their CSOs who are not willing or not able to attend face-to-face services. The current study has nevertheless been able to address changes in treatment and help services for gambling during COVID-19, but also to provide suggestions for longer term developments in addressing gambling harms strategically from a public health framework.
